# Prophylactic Erythropoietin for Neuroprotection in Very Preterm Infants: A Meta-Analysis Update

**DOI:** 10.3389/fped.2021.657228

**Published:** 2021-05-20

**Authors:** Hendrik S. Fischer, Nora J. Reibel, Christoph Bührer, Christof Dame

**Affiliations:** Department of Neonatology, Charité—Universitätsmedizin Berlin, Berlin, Germany

**Keywords:** erythropoietin, meta-analysis, neurodevelopment, neuroprotection, VLBW and ELBW infants

## Abstract

A meta-analysis update of randomized controlled trials investigating recombinant human erythropoietin suggests improved neurodevelopmental outcome in preterm infants. There was substantial heterogeneity, which could be ascribed to a single trial. Exclusion of this trial featuring a high risk of bias abolished heterogeneity and any effects of recombinant human erythropoietin treatment.

## Introduction

Recombinant human erythropoietin (rhEPO) is considered a neuroprotective or neurorestorative drug for the premature brain, because it has been experimentally shown to prevent or to mitigate white matter injury. rhEPO reduces the need for red blood cell transfusions in preterm infants, but only few randomized controlled trials (RCTs) have addressed neurodevelopmental outcomes. Our previous meta-analysis (2017) showed a beneficial effect of prophylactic rhEPO on cognitive scores at 18–24 months' corrected age (1). The recent publication of a large multicenter RCT, *Preterm Erythropoietin Neuroprotection Trial* (PENUT), raises the question as to the robustness of this finding (2).

## Methods

Following the original methodology (1), we aimed to identify all RCTs investigating the effects of prophylactic rhEPO in preterm infants vs. no treatment or placebo and reporting neurodevelopmental outcomes. The literature search, study selection, and data extraction followed the standard search methods of the Cochrane Collaboration (3). The database search was updated on October 11, 2020 (see Supplementary Information A for details). Two authors (H.F. and C.D.) independently searched the databases MEDLINE, Embase, and CENTRAL and used additional information (cross-referencing of previous reviews and trials, expert information, information about ongoing trials of the International Clinical Trials Registry Platform (4) to identify and select studies and extracted the data). The included studies were independently assessed by two study authors (H.F. and N.R.) using the Cochrane risk-of-bias tool for RCTs. Any discrepancies were resolved by a third author (C.B. or C.D.). For all pre-specified outcomes, funnel plots were used to assess for publication bias (3).

The primary outcome was the number of infants with a Mental Development Index (MDI) <70 on the Bayley Scales of Infant Development, second edition (BSID-II), or a composite cognitive score <85 on BSID-III. Equivalence of these cognitive outcome measures was accepted, as a study showed that a composite cognitive score <85 (BSID-III) predicted an MDI <70 (BSID-II) with an overall agreement of 97.3% (5). Secondary outcomes included the number of infants with a Psychomotor Development Index <70 (BSID-II), cerebral palsy, any neurodevelopmental impairment, and a planned subgroup analysis of MDI <70 (BSID-II) or composite cognitive score <85 (BSID-III) in infants of <28 weeks' gestational age.

To include *PENUT*, the timeframe was extended to 18–26 months' corrected age (previously 18–24 months). Moreover, we added (1) a sensitivity analysis that excluded the data from RCTs with a high risk of bias in one or more domains of the Cochrane risk-of-bias assessment tool; (2) exploratory analyses that investigated the effects of prophylactic rhEPO on mortality, on a combined outcome of death or MDI <70 or composite cognitive score <85 and on a combined outcome of death or any neurodevelopmental impairment; and (3) exploratory analyses that investigated the effect of high-dose rhEPO (≥1,000 iU/kg per dose) vs. low- to moderate-dose rhEPO (<1,000 iU/kg per dose) on neurodevelopmental outcome measures. A level of statistical significance of *p* < 0.05 was accepted when testing for subgroup differences.

## Results

The update identified 164 additional records, of which 11 were assessed as full text (Supplementary Figure 1): Six RCTs were excluded because they did not report neurodevelopmental outcomes, and three reported behavioral measures or results of imaging studies at 3.5–6 years (see Supplementary Information B for details). Two more RCTs (2, 6) were included (Table 1). Study details and the complete risk-of-bias assessment are available online (Supplementary Table 1).

**Table 1:** Characteristics of included studies.

**References**	**Year**	***n***	**Gestational age, birth weight**	**Time point of intervention**	**Intervention**	**Recruitment**
Ohls et al. (7)	2004	102	≤ 32 ^0^/_7_, ≤ 1,000 g	24–96 h of age	rhEPO 400 IU/kg IV or SC, 3 times per week until 35 ^0^/_7_ weeks' postmenstrual age	1997–1998
Ohls et al. (8)	2014	53^a^	any GA, ^b^ 500–1,250 g	≤ 48 h of age	rhEPO 400 IU/kg SC, 3 times per week until 35 ^0^/_7_ weeks' postmenstrual age	2006–2010
Natalucci et al. (9)	2016	365	26 ^0^/_7_ to 31 ^6^/_7_, any BW	<3 h of age	rhEPO 3,000 IU/kg IV at <3, 12–18, and 36–42 h of age	2005–2012
Song et al. (10)	2016	613	≤ 32 ^0^/_7_, any BW	<72 h of age	rhEPO 500 IU/kg IV every other day for 2 weeks	2009–2013
Peltoniemi et al. (6)	2017	35	24 ^0^/_7_ to 30 ^0^/_7_, 700–1,500 g	1st day of life	rhEPO 250 IU/kg IV daily from days 1 to 6	1998–2000
Juul et al. (2)	2020	628	24 ^0^/_7_ to 27 ^6^/_7_, any BW	≤ 24 h of age	rhEPO 1,000 IU/kg IV every 48 h for 6 doses, followed by 400 IU SC 3 times per week until 32 ^6^/_7_ weeks' postmenstrual age	2013–2016

*BW, birth weight; GA, gestational age; rhEPO, recombinant human erythropoietin; IU, international units; IV, intravenously; SC, subcutaneously.*

a *This study had three groups: rhEPO (n = 29) vs. placebo (n = 24) vs. darbepoetin (n = 27). The darbepoetin group was not included in the meta-analysis*.

b *Median (interquartile range) GA of included infants: 28 (26–29) weeks; study entry criteria: preterm infants with a birth weight of 500–1,250 g*.

**Figure 1:**
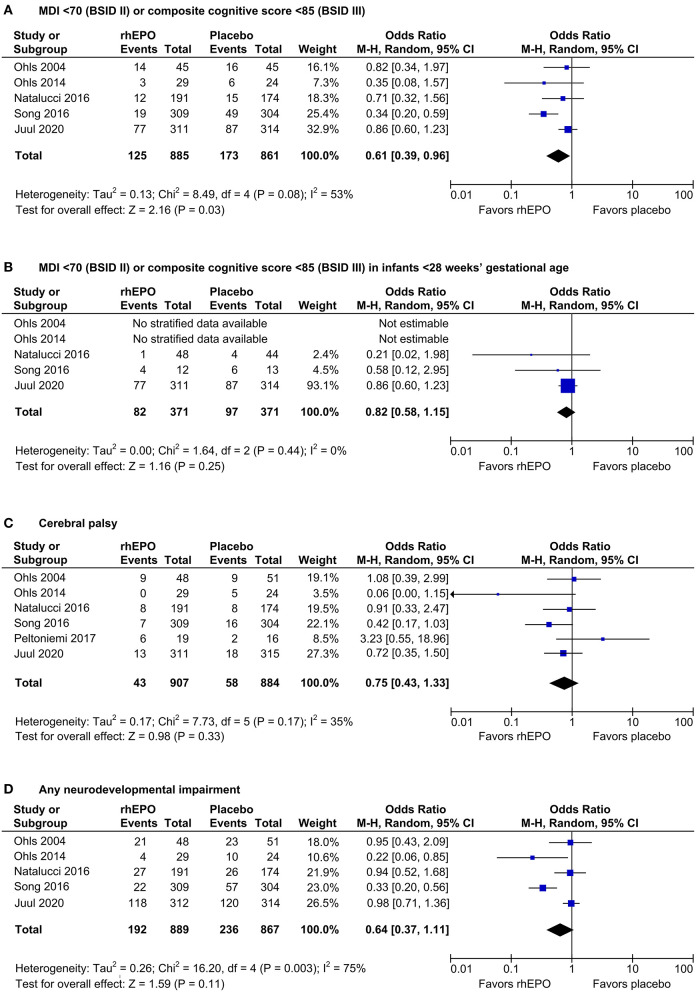
Effects of rhEPO on neurodevelopment at 18–26 months' corrected age. Forest plots show the effects on the number of infants with an MDI <70 (BSID-II) or a composite cognitive score <85 (BSID-III) in all infants (primary outcome, **(A)** and in infants <28 ^1^/_7_ weeks' gestational age (planned subgroup analysis, **(B)**, on cerebral palsy **(C)**, and on any neurodevelopmental impairment **(D)**. M-H, Mantel–Haenszel.

Meta-analysis of the five trials that reported MDI <70 (BSID II) or composite cognitive score <85 (BSID-III) at 18–26 months' corrected age showed a risk reduction from 20 to 14% associated with prophylactic rhEPO (odds ratio 0.61, *p* = 0.03, Figure 1A), corresponding to a number needed to treat of 17. rhEPO had no significant effect on any secondary outcome. Forest plots for outcomes with new data are shown in Figures 1B–D.

The methodological quality of the included RCTs was mostly high, with low or sometimes unclear risk of bias in most domains (Supplementary Table 1). Only one study (10) was categorized as “high risk of bias” in two domains (blinding of participants and personnel and selective reporting). After exclusion of this study, the statistically significant effect of rhEPO on the primary outcome of MDI <70 or a composite cognitive score <85 disappeared (Supplementary Figure 2), as did all indicators of heterogeneity in this analysis (τ^2^ = 0, χ^2^<*df* , *I*^2^ = 0%).

Exploratory analyses showed no effect of rhEPO on mortality, on the combined outcome of death or MDI <70 or a composite cognitive score <85 or on the combined outcome of death or neurodevelopmental impairment (Supplementary Figure 3). The exploratory subgroup analyses showed a beneficial effect of rhEPO on MDI <70 or a composite cognitive score <85 and a borderline beneficial effect on any neurodevelopmental impairment, only in the subgroup of trials that applied low- to moderate-dose rhEPO (Supplementary Figures 4A,D). The tests for subgroup differences showed no differences in the effects of high-dose vs. low- to moderate-dose rhEPO on any neurodevelopmental outcome (Supplementary Figure 4). The assessment of the funnel plots did not reveal major asymmetries (Supplementary Figure 5).

## Discussion

This update continues to indicate a benefit of prophylactic rhEPO by lowering the number of very preterm infants with an MDI of <70 or composite cognitive score of <85 (Figure 1A). However, no effect on MDI <70 or a composite cognitive score <85 could be ascertained in the subgroup of infants born below 28 weeks' gestational age, possibly due to the small number of study patients in this subgroup (Figure 1B). There were no beneficial effects on any secondary outcomes. While significantly improved neurodevelopment at the age of 3.5 to 4 years has been reported in one RCT (11), prophylactic high-dose rhEPO had no effect on cognitive scores at 5 years of age in a large Swiss RCT (12).

The results of the present meta-analysis may have been influenced by publication bias, because most rhEPO RCTs identified in the literature search did not report neurodevelopmental follow-up data. In this setting, the funnel plots were not conspicuous but had a low power to detect publication bias, because a maximum number of 6 RCTs were included in the meta-analysis update (Supplementary Figure 5). Moreover, the uncertainty as to the effects of rhEPO is fostered by the moderate to substantial heterogeneity of the RCTs included in our meta-analysis (*I*^2^ = 53%, Figure 1A; *I*^2^ = 75%, Figure 1D). Statistical heterogeneity and the presumed effect of rhEPO on MDI <70 or a composite cognitive score <85 disappeared after exclusion of a single study (10) featuring increased risk of bias (Supplementary Figure 2A). Discrepancies with the main analysis (Figure 1A) may not necessarily result from bias in the excluded RCT but could reside in differences in the study protocol, in the healthcare environment, or in the population studied.

Looking at the differences between the study protocols, it becomes obvious that quite different dosing regimens of rhEPO were applied (Table 1), depending upon the concept of using rhEPO with the primary intention of neuroprotection/neurorepair vs. prevention of anemia of prematurity or even a combined approach. Admittedly, a subgroup analysis on dosing effects remains very limited due to the heterogeneity of the study protocols. In an exploratory analysis, we compared neurodevelopmental outcomes in RCTs with early high-dose rhEPO (≥1,000 iU/kg per dose) intended for neuroprotection/neurorepair in accordance with experimental data on the transport of rhEPO across the blood–brain barrier (13) vs. low- to moderate-dose rhEPO (<1,000 iU/kg per dose) intended for the prevention of red blood cell (RBC) transfusions. Although the beneficial effect of rhEPO on MDI <70 or a composite cognitive score <85 was preserved in the subgroup meta-analysis of trials that applied low- to moderate-dose rhEPO, the tests for subgroup differences indicated no statistically significant subgroup effect for any outcome (Supplementary Figure 4). Alternatively, the power of this analysis to detect subgroup differences may have been too small, because few trials were included and because less trials contributed data to the high-dose subgroup than to the low-dose subgroup (uneven covariate distribution). Considering the aforementioned uncertainties and the clinical context, we believe that the individual effect of RBC transfusion on neurodevelopment also deserves attention. Interestingly, the *post hoc* analysis of the PENUT trial indicated that each transfusion of RBCs was associated with a decrease in mean cognitive score, motor score, and language score (BSID-III). Moreover, significant negative associations between BSID-III scores, transfusion volume, and donor exposure were observed in the placebo group, but not in the rhEPO group (14). Since an early start of rhEPO therapy significantly reduced the number of RBC transfusions, as shown in the updated Cochrane Review and in the recent PENUT trial (15, 16), rhEPO may still reclaim its value in the patient blood management of very preterm infants.

Finally, a retrospective case–control study suggested that beneficial neurodevelopmental effects of rhEPO may not become apparent prior to preschool age and may be restricted to children who had suffered from intraventricular hemorrhage (17). Thus, we have to await the 5-years neurodevelopmental follow-up data (EpoRepair, NCT02076373) of infants with intraventricular hemorrhage randomized to rhEPO or placebo (18) to decide whether or not rhEPO may have a role in alleviating neurodevelopmental impairment due to a certain pathophysiology of white matter injury.

## Author Contributions

HF designed the meta-analysis, searched for relevant studies, extracted, assessed, and analyzed the data, and drafted the manuscript. NR and CB contributed to data extraction and assessment and critically revised the manuscript. CD conceptualized the meta-analysis, searched for relevant studies, extracted and assessed the data, and drafted the manuscript. All authors critically reviewed and revised the manuscript, approved the final version as submitted, and agreed to be accountable for all aspects of the work.

## Conflict of Interest

The authors declare that the research was conducted in the absence of any commercial or financial relationships that could be construed as a potential conflict of interest.

## Abbreviations

BSD-II, Bayley Scales of Infant Development: second edition
BSD-III, Bayley Scales of Infant Development: third edition
MDI: Mental Development Index
PENUT: Preterm Erythropoietin Neuroprotection Trial
RBC: red blood cell
RCT: randomized controlled trial
rhEPO: recombinant human erythropoietin.
