# Supplementary material for: Prophylactic Erythropoietin for Neuroprotection in Very Preterm Infants: A Meta-Analysis Update
Source: Front Pediatr. 2021 May 20;9:657228. doi: 10.3389/fped.2021.657228 (PMC8173165; doi:10.3389/fped.2021.657228)
Supplement: Supplementary file 1 [file Table_1.docx]

**Supplemental Table 1** Characteristics of included studies, risk of bias assessment

Ohls RK, Ehrenkranz RA, Das A, Dusick AM, Yolton K, Romano E, et al. Neurodevelopmental outcome and growth at 18 to 22 months' corrected age in extremely low birth weight infants treated with early erythropoietin and iron. *Pediatrics*. 2004;114(5):1287-1291.

| Methods | Randomized controlled multicenter trial  Blinding of randomization: yes  Blinding of intervention: yes  Blinding of outcome measurements: yes  Follow up: incomplete |
| --- | --- |
| Participants | 172 extremely low birth weight (ELBW) infants  Inclusion criteria: Infants ≥401 g and ≤1000 g at birth, ≤32 weeks’ gestation, between 24 and 96 hours old at the time of study entry, likely to survive >72 hours, informed consent from a parent or guardian  Exclusion criteria: major congenital anomaly, positive direct antiglobulin test, coagulopathy, clinical seizures, systolic blood pressure >100 mm Hg (in the absence of pressor support), absolute neutrophil count of ≤500/µL |
| Interventions | Erythropoietin group: 400 IU/kg recombinant human erythropoietin (rhEPO) 3x/week intravenously (or subcutaneously when intravenous access was not available), administration started at 24-96 hours of age and stopped at 35 completed weeks’ postmenstrual age  Control group: intravenous sham injections 3x/week, sham subcutaneous injections when intravenous access was not available, adhesive bandage covered true and injection sites |
| Outcomes | Primary outcome: Number of transfusions per infant until discharge  Outcomes at 18 to 22 months’ corrected age: Mental Development Index (MDI) <70 and Psychomotor Development Index (PDI) < 70 as assessed by the Bayley Scales of Infant Development II (BSID-II), cerebral palsy, information about vision status and hearing as obtained by parental reporting and the results of postdischarge ophthalmologic and audiologic evaluations if available, blindness (= no functional vision in both eyes), deafness (= hearing disability requiring amplification), survival with any neurodevelopmental impairment (= MDI <70, or PDI <70, or moderate to severe cerebral palsy, or blindness, or deafness), number of rehospitalizations and transfusions, anthropometric measurements |
| Notes | Supported by grants from the National Institutes of Health, National Institute of Child Health and Human Development, Ortho-Biotech and Schein Pharmaceuticals |

| Risk of Bias: | | |
| --- | --- | --- |
| Bias | Authors’ judgement | Support for judgement |
| Random sequence generation (selection bias) | Low risk | Method of randomization: permuted block method |
| Allocation Concealment (selection bias) | Low risk | Blinding of randomization: yes |
| Blinding of participants and personnel (performance bias) | Low risk | Blinding of intervention: yes (All  caregivers and investigators, except the research nurses, were masked to the treatment assignment) |
| Blinding of outcome assessment (detection bias) | Low risk | Blinding of outcome measurement: yes |
| Incomplete outcome data (attrition bias) | Unclear risk | Complete follow-up: no (Among 142 infants surviving to discharge, 51 of 72 (71%) of the rhEPO-treated and 51 of 70 (73%) of the placebo/control infants were evaluated at 18 to 22 months’ corrected age) |
| Selective reporting (reporting bias) | Unclear risk | Trial not registered |
| Other bias | Low risk | Funding includes a grant from a pharmaceutical company |

Ohls RK, Kamath-Rayne BD, Christensen RD, Wiedmeier SE, Rosenberg A, Fuller J, et al. Cognitive outcomes of preterm infants randomized to darbepoetin, erythropoietin, or placebo. *Pediatrics*. 2014;133(6):1023-1030.

| Methods | Randomized controlled multicenter trial  Blinding of randomization: yes  Blinding of intervention: yes  Blinding of outcome measurements: yes  Follow-up: incomplete |
| --- | --- |
| Participants | 102 preterm infants (rhEPO, n=33; placebo, n= 33; darbepoetin, n=36)  Inclusion criteria: Infants ≥500 g and ≤1250 g at birth, ≤48 h of age, expected to survive the first days of life  Exclusion criteria: Trisomies, significant congenital anomalies, hypertension, seizures, thromboses, hemolytic disease, patients already receiving rhEPO |
| Interventions | Erythropoietin group: 400 U/kg rhEPO 3x/week subcutaneously, initiated at ≤48 h of age and continued until 35 completed weeks’ gestation  Control group: sham doses 3x/week  Darbepoetin group: 10 µg/kg, 1x/week subcutaneously with sham dosing 2x/week |
| Outcomes | Primary outcome at 18 to 22 months’ adjusted age: Cognitive score of the Bayley Scales of Infant Development III (BSID-III)  Secondary outcomes at 18 to 22 months’ adjusted age: Cognitive outcomes <85, <80 and <70 (BSID-III), social-emotional and language scores of the BSID-III, object permanence score as a measure of early working memory, cerebral palsy classified by the Gross Motor Function Classification, visual impairment (= vision requiring correction), hearing impairment (= requirement of a hearing aid, unilateral or bilateral deafness), neurodevelopmental impairment (= BSID-III cognitive score <80, or visual impairment, or hearing impairment, or any cerebral palsy), moderate neurodevelopmental impairment, anthropometric measurements |
| Notes |  |

| Risk of Bias: | | |
| --- | --- | --- |
| Bias | Authors’ judgement | Support for judgement |
| Random sequence generation (selection bias) | Low risk | Method of randomization: computer-generated permuted block method |
| Allocation Concealment (selection bias) | Low risk | Blinding of randomization: yes |
| Blinding of participants and personnel (performance bias) | Low risk | Blinding of intervention: yes (All caregivers and investigators, except the research pharmacists and coordinators administering the study medicine, were masked to the treatment assignment) |
| Blinding of outcome assessment (detection bias) | Low risk | Blinding of outcome measurement: yes |
| Incomplete outcome data (attrition bias) | Unclear risk | Complete follow-up: no (Among 94 surviving infants, 29 of 32 (91%) of the rhEPO-treated and 24 of 30 (80%) of the placebo/control infants completed the follow-up at 18 to 22 months’ corrected age) |
| Selective reporting (reporting bias) | Low risk | ClinicalTrials.gov: NCT00334737 |
| Other bias | Low risk |  |

Natalucci G, Latal B, Koller B, Ruegger C, Sick B, Held L, et al. Effect of Early Prophylactic High-Dose Recombinant Human Erythropoietin in Very Preterm Infants on Neurodevelopmental Outcome at 2 Years: A Randomized Clinical Trial. *JAMA*. 2016;315(19):2079-2085.

| Methods | Randomized controlled multicenter trial  Blinding of randomization: yes  Blinding of intervention: yes  Blinding of outcome measurements: yes  Follow-up: incomplete |
| --- | --- |
| Participants | 450 very preterm infants  Inclusion criteria: Infants born between 26 weeks 0 days’ and 31 weeks 6 days’ gestation, postnatal age less than 3 hours, informed parental consent  Exclusion criteria: genetically defined syndrome, severe congenital malformation adversely affecting life expectancy or neurodevelopment, severe IVH before randomization, a priori palliative care. After one study patient in the rhEPO group died of severe intraventricular hemorrhage, the parents lodged a claim. Subsequently, the Swissmedic mandated that infants with IVH grade 2 or more detected before dose 3 of rhEPO also had to be excluded. |
| Interventions | Erythropoietin group: 3000 IU/kg rhEPO dissolved in 1ml distilled water intravenously before 3 hours, at 12 to 18 hours, and at 36 to 42 hours after birth  Control group: Equivalent volume of isotonic saline (NaCl 0,9%) intravenously before 3 hours, at 12 to 18 hours, and at 36 to 42 hours after birth |
| Outcomes | Primary Outcome at 2 years’ corrected age: MDI as assessed by BSID II  Secondary Outcomes at 2 years’ corrected age: PDI as assessed by BSID-II, cerebral palsy (according to the Gross Motor Function Classification System), severe vision impairment (= blindness or only perception of light or light-reflecting objects), severe hearing impairment (= absence of useful hearing, even with aids, i.e. >90 dB hearing level), severe neurodevelopmental impairment (= MDI <70, or PDI <70, or cerebral palsy with Gross Motor Function Classification System level 3 to 5, or severe hearing impairment, or severe visual impairment), anthropometric measurements  Post hoc exploratory outcomes: MDI <70, PDI <70 |
| Notes | Supported by grants from the Swiss National Science Foundation and the Roche Foundation for Anemia Research |

| Risk of Bias: | | |
| --- | --- | --- |
| Bias | Authors’ judgement | Support for judgement |
| Random sequence generation (selection bias) | Low risk | Method of randomization: block randomization with variable block length by a biometrician |
| Allocation Concealment (selection bias) | Low Risk | Blinding of randomization: yes (randomization list only known to the pharmacist) |
| Blinding of participants and personnel (performance bias) | Low risk | Blinding of intervention: yes (Parents, physicians, nurses, and external statisticians were unaware of treatment allocation) |
| Blinding of outcome assessment (detection bias) | Low risk | Blinding of outcome measurement: yes (All neurodevelopmental assessments were conducted in a blinded manner) |
| Incomplete outcome data (attrition bias) | Unclear risk | Complete follow-up: no (Primary outcome data from 191 of 230 (83%) in the rhEPO group and 174 of 220 (79%) in the placebo group) |
| Selective reporting (reporting bias) | Low risk | ClinicalTrials.gov: NCT00413946 Registered in December 2006, approximately 1 year after enrollment had started.  The outcomes MDI <70 and PDI <70 were added post hoc. |
| Other bias | Low risk | Funding includes a grant from a pharmaceutical company |

Song J, Sun H, Xu F, Kang W, Gao L, Guo J, et al. Recombinant human erythropoietin improves neurological outcomes in very preterm infants. *Ann Neurol*. 2016;80(1):24-34.

| Methods | Randomized controlled two-center trial  Blinding of randomization: yes  Blinding of intervention: no  Blinding of outcome measurements: yes  Follow-up: incomplete |
| --- | --- |
| Participants | 743 very preterm infants  Inclusion criteria: gestational age ≤ 32 weeks, birth weight <1500 g, less than 72 hours of age  Exclusion criteria: Genetic metabolic diseases, congenital abnormalities, polycythemia, infection-induced multiple organ failure, unstable vital signs (multiple organ failure such as respiration or circulation failure), grade 3 or 4 intraventricular hemorrhage (IVH) |
| Interventions | Erythropoietin group: 500U/kg rhEPO intravenously every other day for 2 weeks, administration of first dose within 72 hours after birth  Control group: Equivalent volume of saline with the same treatment procedure as the rhEPO group |
| Outcomes | Primary Outcomes at 18 months’ corrected age: Incidence of MDI <70 using the BSID II (according to the study protocol at ClinicalTrials.gov); incidences of death, of disability and of death or disability (according to the paper)  Secondary Outcomes at 18 months’ corrected age: MDI (BSID II), PDI (BSID-II), cerebral palsy, blindness (= corrected visual acuity <20/200), deafness (= hearing disability that required amplification), moderate to severe disability (= MDI <70, or cerebral palsy, or blindness, or deafness) |
| Notes |  |

| Risk of Bias: | | |
| --- | --- | --- |
| Bias | Authors’ judgement | Support for judgement |
| Random sequence generation (selection bias) | Low risk | Method of randomization: Computer-based random-number generator |
| Allocation Concealment (selection bias) | Low risk | Blinding of randomization: yes (sealed envelopes) |
| Blinding of participants and personnel (performance bias) | High risk | Blinding of intervention: no (the doctors and nurses responsible for the treatment were not blinded, the parents were blinded to the patients’ group allocation) |
| Blinding of outcome assessment (detection bias) | Low risk | Blinding of outcome measurement: yes (The investigators performing the short-term and long-term outcome assessments were blinded to the patients’ group allocation) |
| Incomplete outcome data (attrition bias) | Unclear risk | Complete follow-up: no (of 688 surviving infants, 309 of 345 (90%) of the rhEPO-treated and 304 of 343 (89%) of the placebo/control infants completed the follow-up at 18 months’ corrected age) |
| Selective reporting (reporting bias) | High risk | ClinicalTrials.gov: NCT02036073  Registered in December 2013, 6 months after enrollment was completed. |
| Other bias | Low risk |  |

Peltoniemi OM, Anttila E, Kaukola T, Buonocore G, Hallman M. Randomized trial of early erythropoietin supplementation after preterm birth: Iron metabolism and outcome. *Early Hum Dev*. 2017;109:44-49.

| Methods | Randomized controlled single-center trial  Blinding of randomization: yes  Blinding of intervention: yes  Blinding of outcome measurements: yes  Follow up: incomplete |
| --- | --- |
| Participants | 39 preterm infants  Inclusion criteria: birth weight ≥700 g and ≤1500 g, gestational age between 24 weeks 0 days and 30 weeks 0 days, intra-arterial and intra-venous catheter. Written informed consent  Exclusion criteria: major congenital malformation,  early sepsis, congenital infection, thrombocytopenia, absolute neutrophil count of <500/µl, diastolic blood pressure >60 mm Hg, severe shock, hydrops fetalis |
| Interventions | Erythropoietin group: 250 IU/kg rhEPO (Eprex ®) intravenously in six doses for six consecutive days, starting the first day after birth.  Control group: intravenous isotonic saline as a placebo |
| Outcomes | Primary outcome: severity of acute respiratory disease, measured as the oxygenation index during the first six days of life.  Secondary Outcomes at 2 years corrected age: Total developmental quotient, as assessed by using the Griffiths developmental scale; cerebral palsy, survival without major neurological or neurodevelopmental disorders, height, weight, head circumference. |
| Notes | The study was supported by grants from the Foundation for Pediatric Research, The Alma and K.A. Snellman Foundation (Oulu, Finland), The Arvo ja Lea Ylppö Foundation and the Sigrid Jusélius Foundation (Finland). |

| Risk of Bias: | | |
| --- | --- | --- |
| Bias | Authors’ judgement | Support for judgement |
| Random sequence generation (selection bias) | Unclear risk | Method of randomization: simple randomization using a random number table |
| Allocation Concealment (selection bias) | Low risk | Blinding of randomization: yes |
| Blinding of participants and personnel (performance bias) | Low risk | Blinding of intervention: yes (The study drug and placebo were put into identical syringes, the nurses, doctors and study investigators were blinded) |
| Blinding of outcome assessment (detection bias) | Low risk | Blinding of outcome measurement: yes |
| Incomplete outcome data (attrition bias) | Unclear risk | Complete follow-up: no (among 36 surviving infants, 19 of 20 (95%) of the rhEPO-treated and 16 of 16 (100%) of the control infants were evaluated at 2 years, but only 19 (53% of survivors) received a formal assessment using the Griffiths developmental scale.) |
| Selective reporting (reporting bias) | Unclear risk | Trial not registered |
| Other bias | Low risk |  |

Juul SE, Comstock BA, Wadhawan R, Mayock DE, Courtney SE, Robinson T, et al. A Randomized Trial of Erythropoietin for Neuroprotection in Preterm Infants. *N Engl J Med*. 2020;382(3):233-243.

| Methods | Randomized controlled multi-center trial  Blinding of randomization: yes  Blinding of intervention: yes  Blinding of outcome measurements: yes  Follow up: incomplete |
| --- | --- |
| Participants | 741 extremely preterm infants  Inclusion criteria: gestational age between 24 weeks 0 days and 27 weeks 6 days  Parental consent.  Exclusion criteria: known life-threatening anomalies, chromosomal anomalies, disseminated intravascular coagulopathy, twin-to-twin transfusion, hematocrit level above 65%, hydrops fetalis, known congenital infection |
| Interventions | Erythropoietin group: 1000 IU/kg Erythropoietin intravenously every 48 hours for a total of six doses with initial administration within 24 hours after birth, followed by a maintenance dose of 400 IU per kilogram subcutaneously 3x per week through 32 weeks 6 days of postmenstrual age  Control group: intravenous and subcutaneous sham injections |
| Outcomes | Primary outcome at 22 to 26 months adjusted age: death or severe neurodevelopmental impairment, defined as severe cerebral palsy (GFMCS level >2) or a composite motor score or composite cognitive score <70 (BSID-III)  Secondary Outcomes at 22 to 26 months adjusted age: severe neurodevelopmental impairment, moderate-to-severe neurodevelopmental impairment, defined as moderate cerebral palsy (GMFCS level of 2) or a composite motor score or composite cognitive score <85 (BSID-III); death or moderate-to severe neurodevelopmental impairment |
| Notes | Supported by grants from the National Institute of Neurological Disorders and Stroke.  One study author reports receiving consulting fees from Best Doctors. |

| Risk of Bias: | | |
| --- | --- | --- |
| Bias | Authors’ judgement | Support for judgement |
| Random sequence generation (selection bias) | Low risk | Method of randomization: block randomization, sequences were generated at a central data coordinating center |
| Allocation Concealment (selection bias) | Low risk | Blinding of randomization: yes (randomization sequences were provided directly to the research pharmacy with the use of a trial binder that contained the complete set of trial identification numbers and associated randomization assignments) |
| Blinding of participants and personnel (performance bias) | Low risk | Blinding of intervention: yes (all trial personnel were unaware of the trial-group assignments, with the exception of the staff at the data coordinating center, the site pharmacist, and the staff who administered the maintenance injections) |
| Blinding of outcome assessment (detection bias) | Low risk | Blinding of outcome measurement: yes (examiners were unaware of the participants’ medical histories and the results of brain imaging studies). |
| Incomplete outcome data (attrition bias) | Unclear risk | Among 623 surviving infants, 313 of 413 (76%) of the rhEPO-treated and 315 of 410 (77%) of the control infants completed the follow-up at 22 to 26 months adjusted age. |
| Selective reporting (reporting bias) | Low risk | Trial registered, NCT01378273. |
| Other bias | Low risk |  |
