# Supplementary material for: Prophylactic Erythropoietin for Neuroprotection in Very Preterm Infants: A Meta-Analysis Update
Source: Front Pediatr. 2021 May 20;9:657228. doi: 10.3389/fped.2021.657228 (PMC8173165; doi:10.3389/fped.2021.657228)
Supplement: Supplementary file 2 [file Data_Sheet_1.docx]

**Supplementary Information**

**Part A**

**Search strategies for database searching.**

Our institution provides access to MEDLINE via PubMed (National Library of Medicine, USA), and EMBASE via OvidSP (Wolters Kluwer N.V., The Netherlands), therefore the format of the searches had to be adapted accordingly. The EMBASE search was limited to journals not listed in MEDLINE.

A) Medical Literature Analysis and Retrieval System Online (MEDLINE)

1. Search (((infant, newborn[MeSH Terms]) OR neonat*[tiab]) OR newborn*[tiab]) OR infant*[tiab]
2. Search ((premature[tiab]) OR preterm[tiab]) OR prematurity[tiab]
3. Search (#1) AND #2
4. Search ((((infant, premature[MeSH Terms]) OR retinopathy of prematurity[MeSH Terms]) OR retinopathy of prematurity[tiab]) OR retrolental fibroplasia[tiab]) OR rop[tiab]
5. Search (((((erythropoietin[MeSH Terms]) OR erythropoietin[tiab]) OR erythropoiesis stimulating protein[tiab]) OR epoetin[tiab]) OR rhuepo[tiab]) OR darbepoetin[tiab]
6. Search ((((((randomized controlled trial[Publication Type]) OR clinical trial[Publication Type]) OR random*[tiab]) OR trial[tiab]) OR groups[tiab]) OR blind*[tiab]) OR placebo[tiab]
7. Search (#3) OR #4
8. Search ((#5) AND #6) AND #7
9. Search ((((((((#1) OR #4) OR child[MeSH Terms]) OR toddler*[tiab]) OR preschool[tiab]) OR pre-school[tiab]) OR child[tiab]) or paediatric[tiab]) OR pediatric[tiab]
10. Search ((((((((((((((auditory[tiab]) OR cogniti*[tiab]) OR hearing[tiab]) OR intelligence[tiab]) OR language[tiab]) OR mental[tiab]) OR motility[tiab]) OR motor[tiab]) OR neurological[tiab]) OR neuromotor[tiab]) OR psychological[tiab]) OR psychomotor[tiab]) OR speech[tiab]) OR vision[tiab]) OR visual[tiab]
11. Search ((((((((assessment[tiab]) OR development*[tiab]) OR impairment[tiab]) OR index[tiab]) OR maturation[tiab]) OR outcome[tiab]) OR performance[tiab]) OR test[tiab]) OR testing[tiab]
12. Search (((neuroprotection[MeSH Terms]) OR psychological test[MeSH Terms]) OR psychomotor performance[MeSH Terms]) OR cerebral palsy[MeSH Terms]
13. Search (child development[MeSH Terms])
14. Search ((#9) AND #10) AND #11
15. Search (#9) AND #12
16. Search ((#13) OR #14) OR #15
17. Search ((#6) OR follow-up studies[MeSH]) OR follow-up[tiab]
18. Search ((#16) AND #17) AND #5
19. Search (#8) OR #18

B) Excerpta Medica Database (EMBASE)

1. (neonat$ or newborn? or infant$).tw. or exp newborn/
2. (premature or preterm or prematurity).tw.
3. 1 and 2
4. exp prematurity/ or exp retrolental fibroplasia/ or (retinopathy of prematurity or retrolental fibroplasia or rop).tw.
5. exp erythropoietin/ or exp recombinant erythropoietin/ or exp novel erythropoiesis stimulating protein/ or (erythropoietin or erythropoiesis stimulating protein or epoetin or rhuepo or darbepoetin).mp.
6. (random$ or groups or blind$ or placebo).tw. or (clinical trial).mp. or exp clinical trial/ or exp health care quality/
7. 3 or 4
8. 5 and 6 and 7
9. 1 or 4 or (toddler? or preschool or pre-school or child or paediatric or pediatric).tw. or exp child/
10. (auditory or cogniti$ or hearing or intelligence or language or mental or motility or motor or neurological or neuromotor or psychological or psychomotor or speech or vision or visual).tw.
11. (assessment or development$ or impairment or index or maturation or outcome or performance or test or testing).tw.
12. exp neuroprotection/ or exp mental function assessment/ or exp neuropsychological test/ or exp mental function/ or exp brain development/ or exp cerebral palsy/
13. exp postnatal development/
14. 9 and 10 and 11
15. 9 and 12
16. 13 or 14 or 15
17. 6 or follow-up.tw. or exp follow up/
18. 5 and 16 and 17
19. 8 or 18
20. limit 19 to exclude Medline journals

C) Cochrane Central Register of Controlled Trials (CENTRAL)

1. (neonat*):ti,ab OR (newborn?):ti,ab OR (infant?):ti,ab OR [mh “infant, newborn“]
2. (premature):ti,ab OR (preterm):ti,ab OR (prematurity):ti,ab
3. {AND #1-#2}
4. (“retinopathy of prematurity”):ti,ab OR (“retrolental fibroplasia”):ti,ab OR (rop):ti,ab OR [mh “retinopathy of prematurity“] OR [mh“infant, premature“]
5. (erythropoietin):ti,ab OR (“erythropoiesis stimulating protein”):ti,ab OR (epoetin):ti,ab OR (rhuepo):ti,ab OR (darbepoetin):ti,ab OR [mh “erythropoietin“]
6. {#3 OR #4}
7. {AND #5-#6}
8. #1 OR #4 OR (toddler):ti,ab OR (preschool):ti,ab OR (pre-school):ti,ab OR (child):ti,ab OR (paediatric):ti,ab OR (pediatric):ti,ab OR [mh “child”]
9. (auditory):ti,ab OR (cogniti*):ti,ab OR (hearing):ti,ab OR (intelligence):ti,ab OR (language):ti,ab OR (mental):ti,ab OR (motility) :ti,ab OR (motor):ti,ab OR (neurologic):ti,ab OR (neuromotor):ti,ab OR (psychological):ti,ab OR (psychomotor):ti,ab OR (speech):ti,ab OR (vision):ti,ab OR (visual):ti,ab
10. (assessment):ti,ab OR (development):ti,ab OR (impairment):ti,ab OR (index):ti,ab OR (maturation) :ti,ab OR (outcome):ti,ab OR (performance):ti,ab OR (test):ti,ab OR (testing):ti,ab
11. [mh “neuroprotection”] OR [mh “psychological test”] OR [mh “psychomotor performance”] OR [mh “cerebral palsy”]
12. [mh “child development”]
13. {AND #8-#10}
14. #8 AND #11
15. {OR 12-#14}
16. #5 AND #15
17. #7 OR #16

**Part B**

**References to new studies excluded from this meta-analysis update**

^a)^ Gasparovic C, Caprihan A, Yeo RA, Phillips J, Lowe JR, Campbell R, et al. The long-term effect of erythropoiesis stimulating agents given to preterm infants: a proton magnetic resonance spectroscopy study on neurometabolites in early childhood. *Pediatr Radiol.* (2018) 48:374-82. doi: 10.1007/s00247-017-4052-1.

Hosseini M, Azampour H, Raeisi S, Behtari M, Valizadeh H, Saboohi R. The effects of enteral artificial amniotic fluid-containing erythropoietin on short term outcomes of preterm infants. *Turk J Pediatr.* (2019) 61:392-8. doi: 10.24953/turkjped.2019.03.011.

^b)^ Lowe JR, Rieger RE, Moss NC, Yeo RA, Winter S, Patel S, et al. Impact of Erythropoiesis-Stimulating Agents on Behavioral Measures in Children Born Preterm. *J Pediatr.* (2017) 184:75-80.e1. doi: 10.1016/j.jpeds.2017.01.020.

Omar OM, Massoud MN, Ghazal H, Hassouna H, Somaa MF. Effect of enteral erythropoietin on feeding-related complications in preterm newborns: A pilot randomized controlled study. *Arab J Gastroenterol.* (2020) 21:37-42. doi: 10.1016/j.ajg.2020.01.001.

^c)^ Phillips J, Yeo RA, Caprihan A, Cannon DC, Patel S, Winter S, et al. Neuroimaging in former preterm children who received erythropoiesis stimulating agents. *Pediatr Res.* (2017) 82:685-90. doi: 10.1038/pr.2017.130.

Qiao L, Tang Q, Zhu W, Zhang H, Zhu Y, Wang H. Effects of early parenteral iron combined erythropoietin in preterm infants: A randomized controlled trial. *Medicine (Baltimore)* (2017) 96:e5795. doi: 10.1097/MD.0000000000005795.

Sharafutdinova DR, Balashova EN, Ionov OV, Kirtbaya AR, Golubtsova JM, Zubkov VV et al. [The recombinant human erythropoietin therapy for extremely and very low birth weight infants.] *Pediatric hematology/oncology and immunopathology* (2019) 18:75‒82. doi: 10.24287/1726-1708-2019-18-2-75-82

Wang Y, Song J, Sun H, Xu F, Li K, Nie C, et al. Erythropoietin prevents necrotizing enterocolitis in very preterm infants: a randomized controlled trial. *J Transl Med.* (2020) 18:308. doi: 10.1186/s12967-020-02459-w.

Yang SS, Xu FL, Cheng HQ, Xu HR, Yang L, Xing JY, et al. [Effect of early application of recombinant human erythropoietin on white matter development in preterm infants]. *Zhongguo Dang Dai Er Ke Za Zhi* (2018) 20:346-51. doi: 10.7499/j.issn.1008-8830.2018.05.002.

__________________________________________________________________________________

Specific Reasons for exclusion:

1. follow-up study of Ohls et al. *Pediatrics* 2014, reported neuroimaging results (MRI) at 3.5–4 years of age
2. follow-up study of Ohls et al. *Pediatrics* 2014, reported behavioral measures at 3.5-4 years of age
3. follow-up study of Ohls et al. *Pediatrics* 2014, reported neuroimaging results (proton magnetic resonance spectroscopy) at 4-6 years of age

The other 6 RCTs were excluded because they did not report neurodevelopmental outcomes.
