# Supplementary material for: Prophylactic Erythropoietin for Neuroprotection in Very Preterm Infants: A Meta-Analysis Update
Source: Front Pediatr. 2021 May 20;9:657228. doi: 10.3389/fped.2021.657228 (PMC8173165; doi:10.3389/fped.2021.657228)
Supplement: Supplementary file 3 [file Data_Sheet_2.docx]

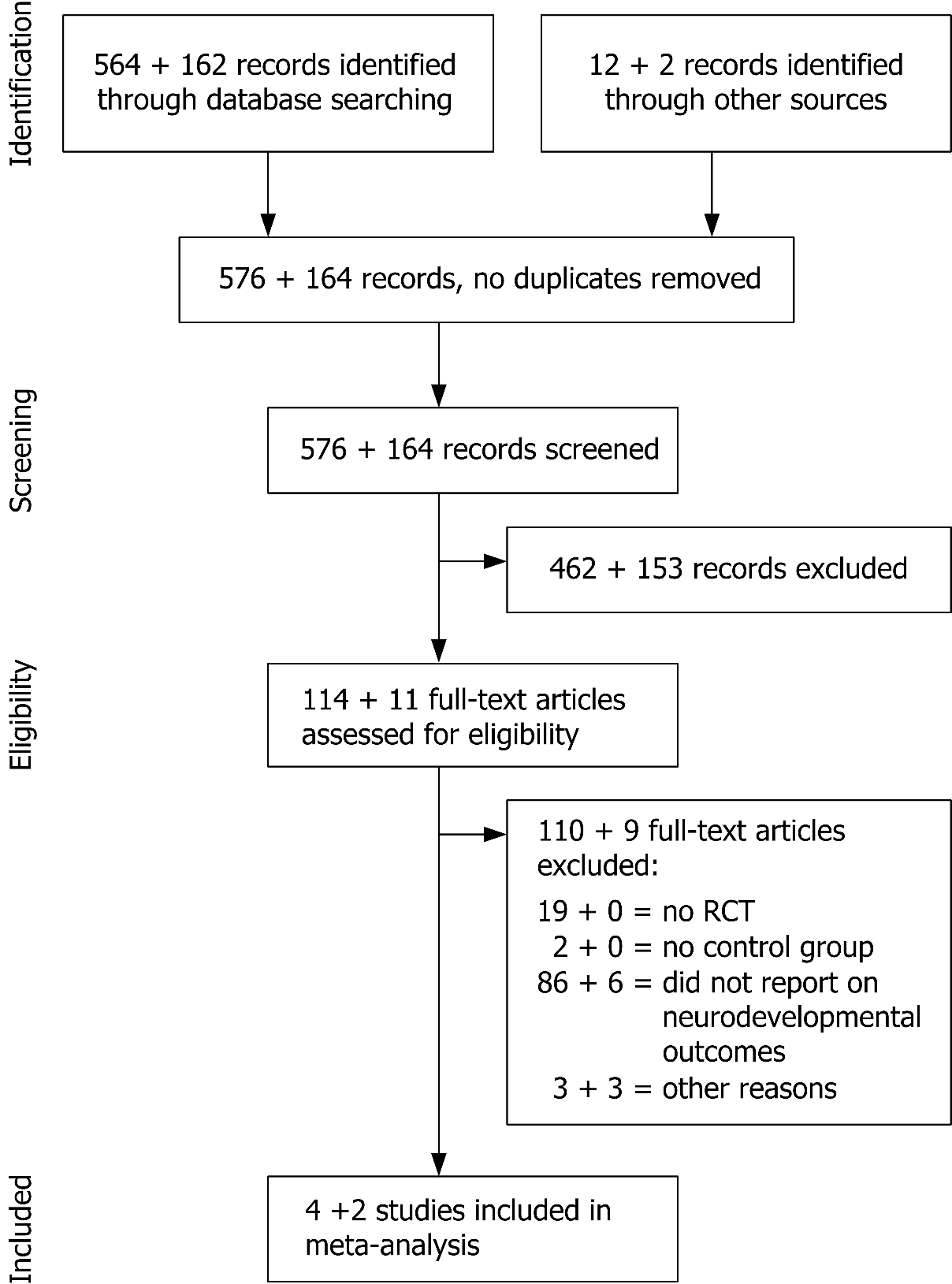


**Supplemental Figure S1.** Preferred Reporting Items for Systematic Reviews and Meta-Analyses (PRISMA) flow diagram. At each stage, the first number refers to the previous version of this meta-analysis, whereas the second number represents the newly identified records.
