# Supplementary material for: Prophylactic Erythropoietin for Neuroprotection in Very Preterm Infants: A Meta-Analysis Update
Source: Front Pediatr. 2021 May 20;9:657228. doi: 10.3389/fped.2021.657228 (PMC8173165; doi:10.3389/fped.2021.657228)
Supplement: Supplementary file 5 [file Data_Sheet_4.docx]

**
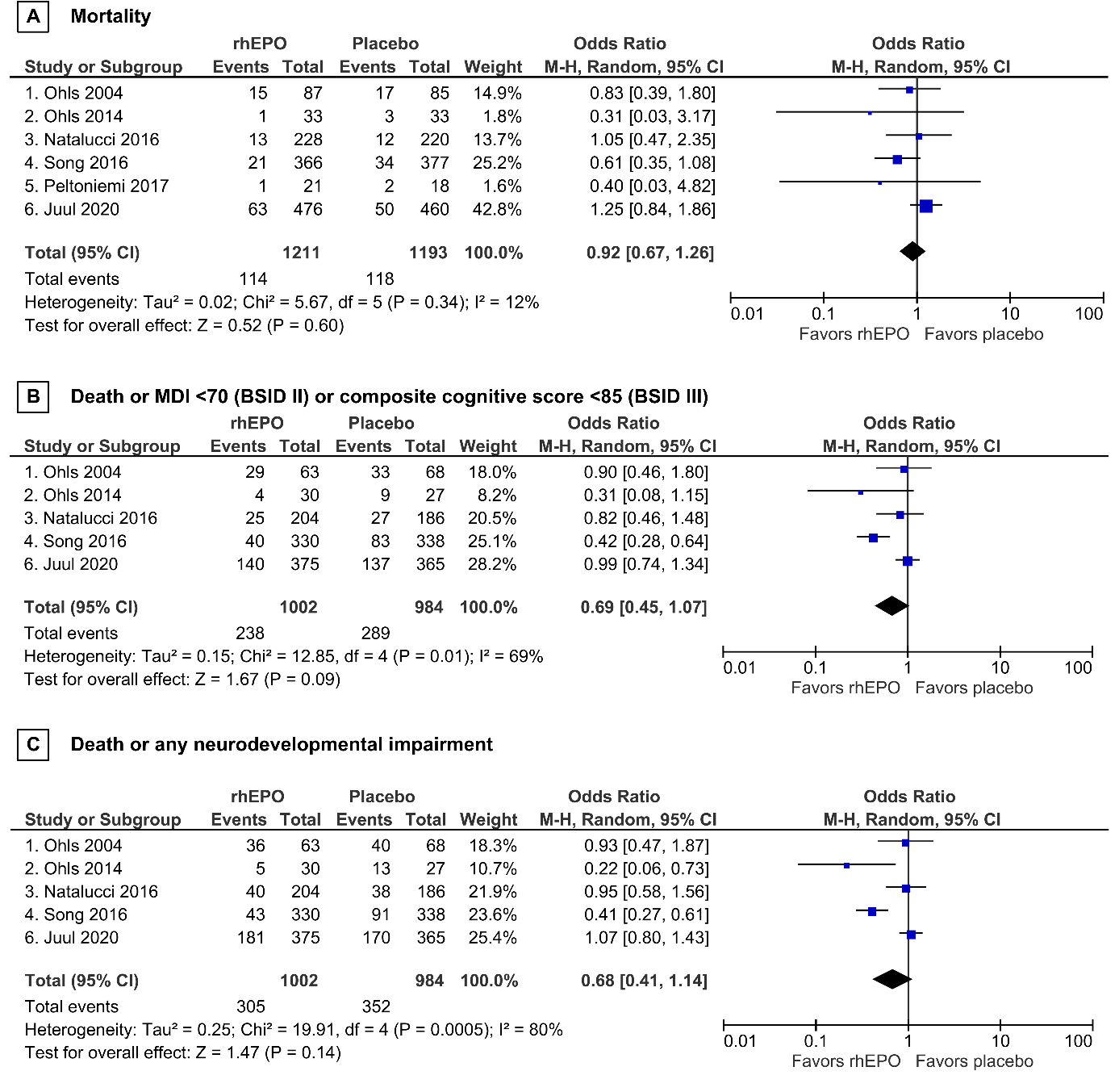
**

**Supplemental Figure S3.** Effects of prophylactic rhEPO on mortality (A), on the combined outcome of death or MDI <70 (BSID II) or composite cognitive score <85 (BSID III) (B), and on the combined outcome of death or any neurodevelopmental impairment at 18-26 months’ corrected age (C). M-H, Mantel–Haenszel.
