# Supplementary material for: Prophylactic Erythropoietin for Neuroprotection in Very Preterm Infants: A Meta-Analysis Update
Source: Front Pediatr. 2021 May 20;9:657228. doi: 10.3389/fped.2021.657228 (PMC8173165; doi:10.3389/fped.2021.657228)
Supplement: Supplementary file 6 [file Data_Sheet_5.docx]

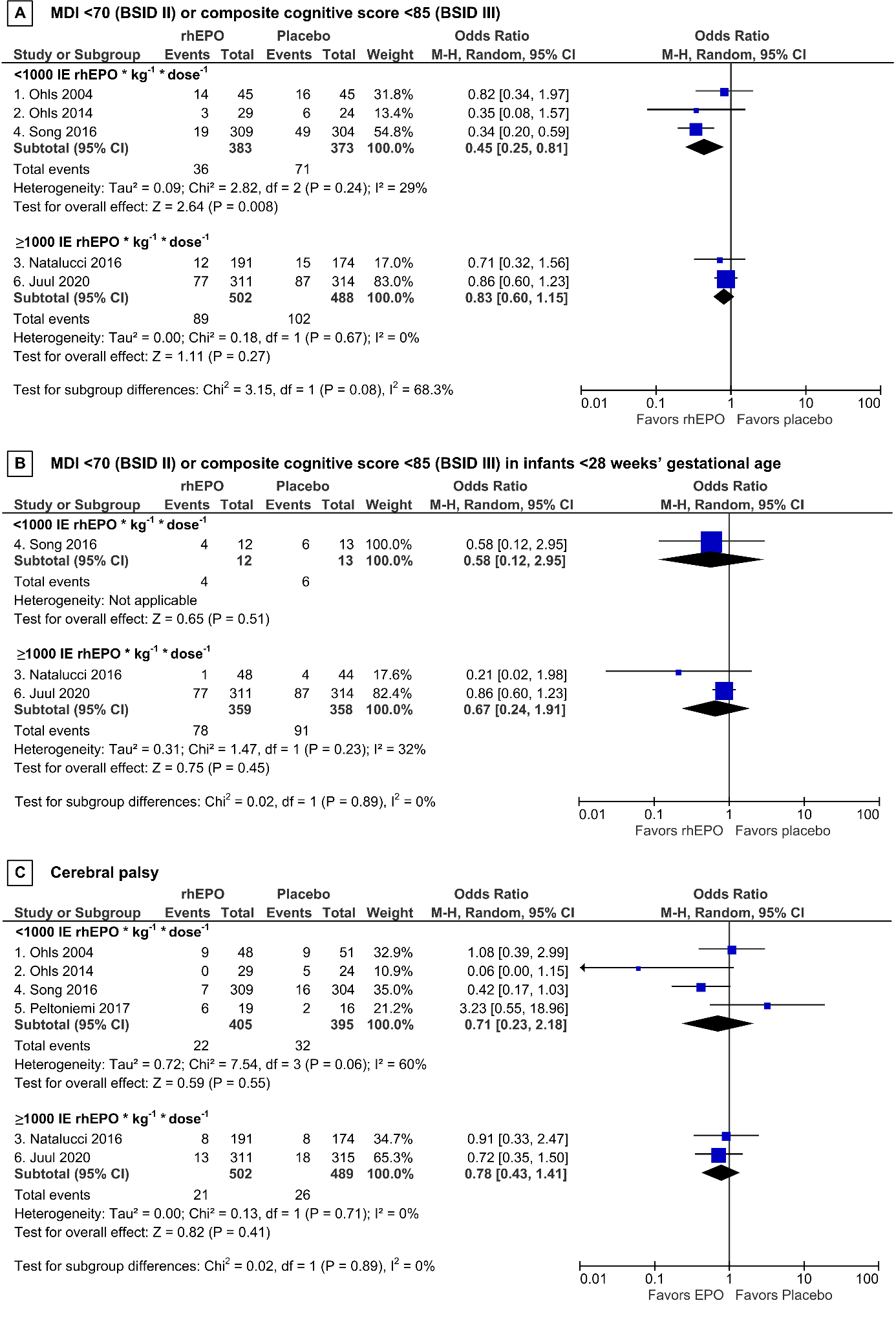


**
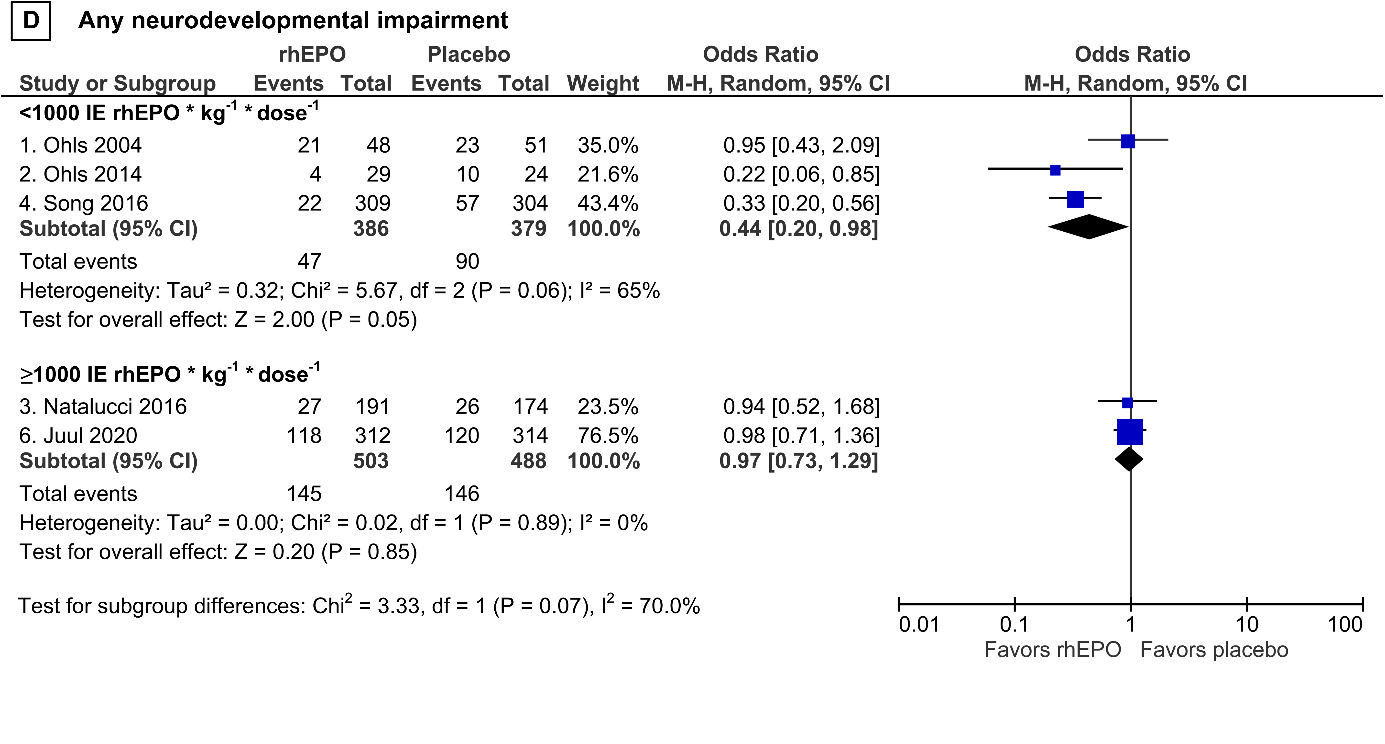
**

**Supplemental Figure S4.** Effects of rhEPO on neurodevelopment at 18-26 months’ corrected age. Subgroup analyses show the effects of high-dose rhEPO and of low- to moderate-dose rhEPO on the number of infants with an MDI <70 (BSID-II) or composite cognitive score <85 (BSID-III) in all infants (primary outcome, A), and in infants <28 weeks’ gestational age (B), on cerebral palsy (C) and on any neurodevelopmental impairment (D). M-H, Mantel–Haenszel.
