# Supplementary material for: Prophylactic Erythropoietin for Neuroprotection in Very Preterm Infants: A Meta-Analysis Update
Source: Front Pediatr. 2021 May 20;9:657228. doi: 10.3389/fped.2021.657228 (PMC8173165; doi:10.3389/fped.2021.657228)
Supplement: Supplementary file 7 [file Data_Sheet_6.docx]

**
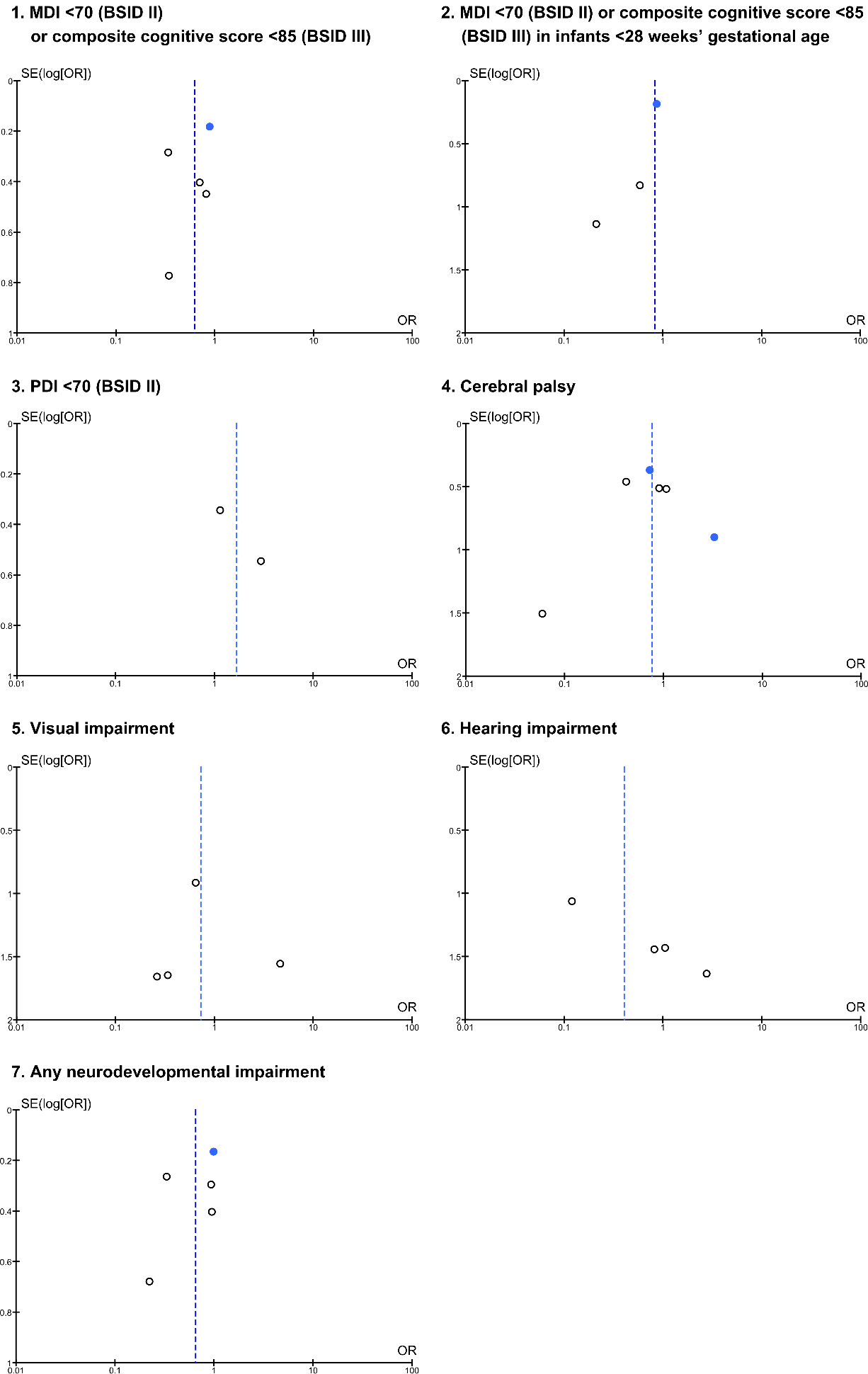
**

**Supplemental Figure S5.** Funnel plots. Odds ratios of the treatment effects of rhEPO plotted against SE (log(OR)) Empty dots refer to the previous version of this meta-analysis, whereas full dots represent data from the newly identified RCTs.
